# Looking the Part: Social Status Cues Shape Race Perception

**DOI:** 10.1371/journal.pone.0025107

**Published:** 2011-09-26

**Authors:** Jonathan B. Freeman, Andrew M. Penner, Aliya Saperstein, Matthias Scheutz, Nalini Ambady

**Affiliations:** 1 Department of Psychology, Tufts University, Medford, Massachusetts, United States of America; 2 Department of Sociology, University of California, Irvine, California, United States of America; 3 Department of Sociology, Stanford University, Stanford, California, United States of America; 4 Department of Computer Science, Tufts University, Medford, Massachusetts, United States of America; 5 Department of Psychology, Stanford University, Stanford, California, United States of America; University College London, United Kingdom

## Abstract

It is commonly believed that race is perceived through another's facial features, such as skin color. In the present research, we demonstrate that cues to social status that often surround a face systematically change the perception of its race. Participants categorized the race of faces that varied along White–Black morph continua and that were presented with high-status or low-status attire. Low-status attire increased the likelihood of categorization as Black, whereas high-status attire increased the likelihood of categorization as White; and this influence grew stronger as race became more ambiguous (Experiment 1). When faces with high-status attire were categorized as Black or faces with low-status attire were categorized as White, participants' hand movements nevertheless revealed a simultaneous attraction to select the other race-category response (stereotypically tied to the status cue) before arriving at a final categorization. Further, this attraction effect grew as race became more ambiguous (Experiment 2). Computational simulations then demonstrated that these effects may be accounted for by a neurally plausible person categorization system, in which contextual cues come to trigger stereotypes that in turn influence race perception. Together, the findings show how stereotypes interact with physical cues to shape person categorization, and suggest that social and contextual factors guide the perception of race.

## Introduction

Individuals categorize others' race—and many other social category memberships—using physical features of the face [1], [2], [3]. Yet, there is growing evidence that race can be cued by nonphysical characteristics as well. A mere mention of the words “welfare” or “inner-city,” for example, can make race salient in policy debates [4], [5]. Moreover, knowledge of whether a person has been incarcerated, impoverished, or has died as a result of a homicide can all influence the race to which a person is assigned [6], [7]. This growing body of sociological work provides an empirical basis for the longstanding claim in the social sciences that race is socially constructed and imbued meaning, in part, by social factors (e.g., [8]). Converging with research in experimental psychology [9], [10], such work suggests that whether a person is categorized as White or Black might depend not just on his or her physical features, but also on a variety of social stereotypes held by perceivers. In the present research, we incorporate these insights with a recent theoretical approach to person categorization and neural network model of its underlying cognitive processing. We bring these together to provide an account of how stereotypes interact with physical cues to shape the perception of another's race.

Although prior research has examined how biographical knowledge about a person may influence how that person is race-categorized [5], [6], [7], [9], [11], when initially interacting with others perceivers generally lack such knowledge. Instead, perceivers' initial categorizations rely only on minimal physical cues. Another's facial and hair cues (e.g., skin color, texture) provide diagnostic information used for race categorization [2], [12]. Yet cues contextualizing the face that are not generally considered race-diagnostic [13] might also play an important role. Specifically, if certain contextual cues are stereotypically associated with certain race categories, the processing of those cues could potentially alter the perception of a face's race. This would be due to the interactive nature of the underlying person categorization process itself.

### Dynamic Interactive Nature of Person Categorization

A recent approach to person categorization views it as an ongoing process where multiple information sources—both bottom-up facial cues and top-down stereotyped expectations—interact over time to stabilize onto an ultimate categorization, e.g., White or Black [14]. This is because person categorization, as implemented in a human brain, might involve continuous changes in a pattern of neuronal activity [15], [16], [17]. As such, early in processing, representations of a face's race might tend to be partially consistent with multiple categories (both White and Black) because the initial rough “gist” of the face partially supports both categories. As more information accumulates, the pattern of neuronal activity would gradually sharpen into an increasingly confident representation (e.g., White), while other competing, partially-active representations (e.g., Black) would be pushed out [16], [18], [19]. During the hundreds of milliseconds it takes for the neuronal activity to achieve a stable pattern (∼100% White or ∼100% Black), both visual processing of the face as well as top-down stereotypes might gradually exert their influences, jointly determining the pattern to which the system gravitates [14], [17], [20], [21]. This approach proposes, therefore, that race categorization involves ongoing competition between partially-active White and Black categories. Further, the competition is gradually weighed in on by both bottom-up facial cues as well as top-down stereotypes, until a stable categorization is achieved. This categorization would reflect a compromise between how a face “actually” appears and the stereotyped expectations dictating how that face “should” appear [14].

One potential trigger of these stereotypes could be the contextual cues that often surround a face in the real world, such as attire. Once activated via contextual cues, stereotypes could alter the processing of a face's race. Businesspeople, for example, are stereotypically associated as high-status, whereas janitors are associated as low-status. However, White individuals, too, are associated as high-status, whereas Black individuals are associated as low-status [22]. Due to this overlap in the stereotypes associated with both race and occupation categories, contextual cues to occupation (e.g., business attire) might potentially activate stereotypes (e.g., high-status) that then exert top-down pressure on the race categorization process, swaying it toward the associated category (e.g., White). For example, business attire could activate high-status stereotypes that then gradually push the race-category competition—primarily being driven by visual processing of the face—more toward the White category. Conversely, janitor attire could activate low-status stereotypes that then gradually push the race-category competition more toward the Black category. Race categorization, therefore, could be driven by both the bottom-up processing of facial features, and top-down stereotypes activated by contextual cues, which mutually constrain one another before a stable categorization is achieved [14].

One implication of this tight exchange between bottom-up and top-down forces we theorize is that, as one force gets weaker, the other force is given sway to exert an increasingly stronger influence on categorization. Thus, as race-specifying facial cues become increasingly ambiguous, the bottom-up ambiguity opens the door wider and wider to stereotypes' top-down influences. This is important because perceivers in the real-world regularly encounter racially ambiguous faces (e.g., multiracial individuals). Despite their ambiguity, however, perceivers rapidly resolve such faces into monoracial categories, such as White or Black [23]. We thus explored whether racial ambiguity might affect the degree to which contextual status cues are able to shift race categorization.

The account we have proposed here makes a number of predictions regarding how contextual cues and the stereotypes they trigger might work to alter race perception. In some cases, especially when race is ambiguous, contextual cues to occupation (business/janitor attire) could trigger stereotypes (high-status/low-status) that exert a strong top-down influence, pushing categorization toward the race category with which they are stereotypically associated (White/Black). Thus, high-status cues would tend to elicit White categorizations, whereas low-status cues would tend to elicit Black categorizations. In other cases, especially when race is more clear-cut, contextual cues would still, nevertheless, exert an influence. Even when perceivers ultimately categorize a janitor-attired face as White or a business-attired face as Black, the status cues would affect the categorization process. The cues would lead perceivers to partially, simultaneously categorize the face as the other race, due to status cues tied to that race, before stabilizing onto their ultimate categorization. Thus, sometimes the influences of status cues would be so strong as to alter an ultimate perceptual outcome; other times they would only subtly alter the perceptual process by temporarily shifting race perception.

### The Present Research

**Figure 1:**
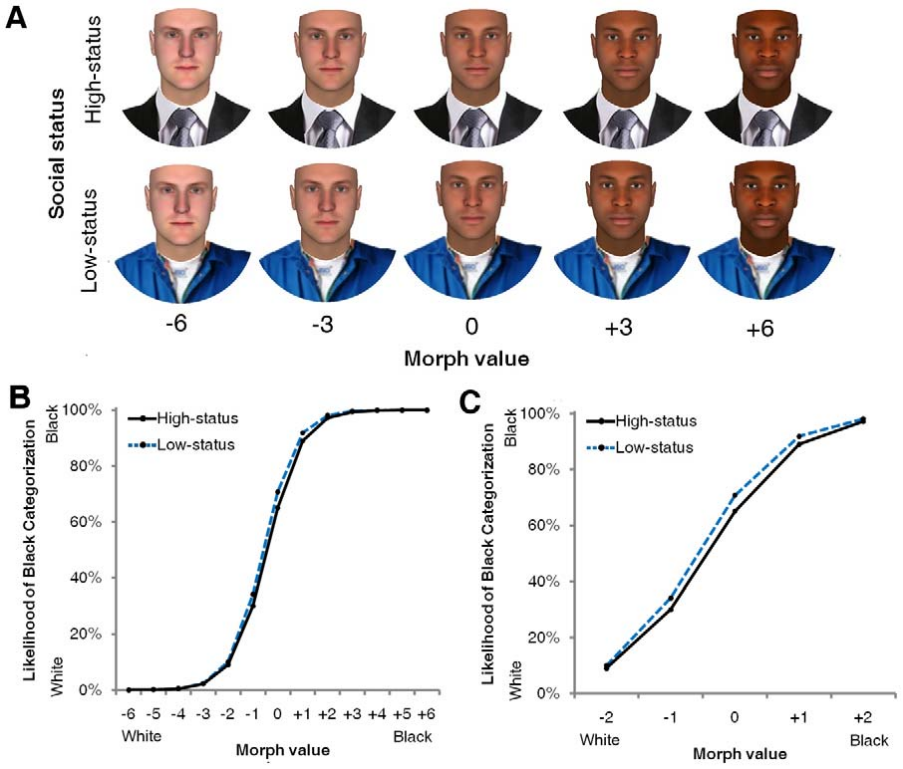
The influence of status cues on race categorization. (**A**) Sample stimuli. A high-status or low-status cue was affixed to 13-point morph continua, where race was varied from White (−6) to Black (+6). (**B**) The likelihood of Black categorization is plotted as a function of morph values, separately for faces with high-status versus low-status attire (Experiment 2). Note the canonical sigmoidal shape of the curves, consistent with the categorical perception of race [30]. Also note that the strongest influences of the status cue are in the middle of the continuum (most clearly shown in Fig. 1C). (**C**) The same plot as in Fig. 1B, except here zooming in on the middle of the morph continuum, where race is most ambiguous.

We tested these ideas in 3 studies. First, in Experiment 1, participants categorized the race of facial morphs that varied from White to Black, contextualized by either business or janitor attire (see Fig. 1A). We predicted that business attire would raise the likelihood of White categorization whereas janitor attire would raise the likelihood of Black categorization. Further, these influences would grow stronger as a face becomes more racially ambiguous. In Experiment 2, participants made these same categorizations, but in a computer mouse-tracking paradigm where their hand trajectories were recorded while traveling toward potential responses on the screen [24]. This mouse-tracking paradigm permits an inspection of the real-time categorization process and the parallel activation of multiple social categories (e.g., [18], [19]). We predicted, again, that high-status business attire would elicit White categorizations and low-status janitor attire would elicit Black categorizations. Even when a status cue would not influence an ultimate categorization response, however, we predicted that it would still lead perceivers to partially, simultaneously activate the other race category with which it is associated. Such a partial parallel activation of the other race category—due to status cues tied to that category—would be evidenced by a partial attraction in participants' hand movements toward the other category response (e.g., Black) before clicking their final response (e.g., White). Lastly, in a final study, we computationally accounted for the results of Experiments 1 and 2 using a neural network model of person categorization. Together, the present studies aimed to test how stereotypes interact with physical cues to shape—sometimes wholesale and other times only in part—the perception of another's race.

## Results

In Experiments 1 and 2, participants were instructed to categorize faces as White or Black as quickly and accurately as possible. The faces varied along 13-point White–Black morph continua, and they were affixed to either high-status business attire or low-status janitor attire (Fig. 1A; see Materials and Methods). In Experiment 1, participants pressed a keyboard button to indicate their response. In Experiment 2, participants moved the computer mouse from a “Start” button at the bottom-center of the screen to click on a “White” or “Black” response button, located in the top-left and top-right corners. In all regression analyses, we adopted a generalized estimating equation (GEE) approach [25] and report unstandardized regression coefficients.

### Experiment 1

First, we regressed perceived race (0 = White, 1 = Black) onto morph values (−0.5 = most prototypically White [morph −6], 0.5 = most prototypically Black [morph +6]), status cue (−0.5 = high-status, 0.5 = low-status), and the interaction (using logistic regression). Expectedly, as morph values rose from White to Black, the likelihood of Black categorization increased, *B* = 13.47, *p*<.0001, *z* = 19.01, confirming our morphing manipulation. Status cues, however, also influenced categorization. A low-status cue raised the likelihood of Black categorization relative to a high-status cue, which raised the likelihood of White categorization, *B* = 0.18, *p*<.05, *z* = 2.10. The interaction was not significant, *B* = 0.96, *p* = .12, *z* = 1.57.

To directly examine whether racial ambiguity may have moderated the influence of status cues on categorization, we generated an index of racial ambiguity by converting morph values into absolute values, multiplying by −1, and centering around 0: −0.5 = most prototypical (morph ±6) to 0.5 = most ambiguous (morph 0). We regressed perceived race onto racial ambiguity, status cue, and the interaction (using logistic regression). Increases in racial ambiguity overall led to increases in the likelihood of Black categorization, *B* = 0.63, *p*<.0001, *z* = 4.26. This was due to an overall bias of categorizing racially ambiguous faces as Black rather than White, as the most ambiguous face (morph 0) had a likelihood of Black categorization in the 60–70% range, rather than 50%. This is consistent with prior work on hypodescent (the tendency to assign individuals of mixed heritage to the social group of lowest status) in race categorization (e.g., [23]). As in the previous analysis, status cues influenced categorization as well, with a low-status cue raising the likelihood of Black categorization and vice-versa for a high-status cue, *B* = 0.05, *p*<.05, *z* = 2.06. More importantly, a significant interaction indicated that the influences of status cues were exacerbated as racial ambiguity increased, *B* = 0.25, *p*<.01, *z* = 2.90.

Thus, contextual status cues shaped race perception, and ambiguity moderated their ability to exert an influence. Although status cues affected a considerable number of categorizations, there were also many categorizations that remained unaffected by status cues. The account we propose, however, argues that such seemingly unaffected categorizations are, in fact, still subtly influenced by those cues. This is because the processing of status cues would always partially weigh in on the categorization process, as described above. Thus, even when a face with low-status attire is categorized as White or a face with high-status attire is categorized as Black, the status cue would still trigger the partial parallel activation of the other race category with which it is associated, thereby temporarily altering race perception. We tested this in Experiment 2 via mouse-tracking.

### Experiment 2

**Figure 2:**
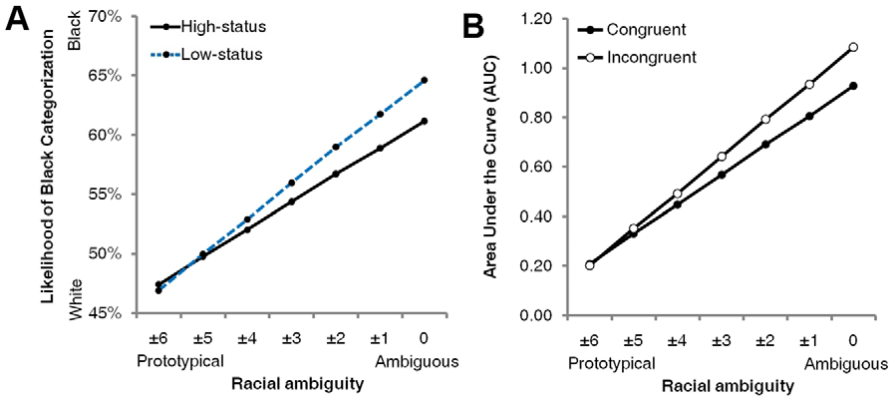
Racial ambiguity's moderation of the influence of status cues. (**A**) The likelihood of Black categorization is plotted as a function of racial ambiguity, separately for faces surrounded by high-status versus low-status attire (Experiment 2). (**B**) The degree of the hand's attraction toward the opposite race-category response (indexed by trajectory curvature) is plotted as a function of racial ambiguity, separately for trials where the categorization response was stereotypically congruent versus incongruent with the status cue (Experiment 2).

Using the coding scheme of Experiment 1, we regressed perceived race onto morph value, status cue, and the interaction. As morph values rose from White to Black, the likelihood of Black categorization increased, *B* = 18.05, *p*<.0001, *z* = 15.22. Further, a low-status cue raised the likelihood of Black categorization relative to a high-status cue, which raised the likelihood of White categorization, *B* = 0.26, *p*<.05, *z* = 2.42 (Figs. 1B and 1C). The interaction was not significant, *B* = 0.85, *p* = .41, *z* = 0.82. As in Experiment 1, morph values were recoded into a racial ambiguity scale to examine ambiguity's moderation of status cues' influence on categorization. As found in Experiment 1, racial ambiguity overall increased the likelihood of Black categorization, *B* = 0.64, *p* = .0001, *z* = 3.88 (see Experiment 1 for explanation). As in the previous analysis, a low-status cue increased the likelihood of Black categorization and vice-versa for a high-status cue, *B* = 0.06, *p*<.05, *z* = 2.29. Further, a significant interaction indicated that these influences of the status cue were exacerbated by increasing levels of racial ambiguity, *B* = 0.17, *p* = .05, *z* = 1.92 (Fig. 2A). These results replicate those of Experiment 1.

For mouse-trajectory analyses, trials were coded as congruent or incongruent based on whether the categorization response was stereotypically congruent vs. incongruent with the status cue. Thus, trials where a face with high-status attire was categorized as White or a face with low-status attire was categorized as Black were coded as congruent; trials where a face with low-status attire was categorized as White or a face with high-status attire was categorized as Black were coded as incongruent.

Trajectories' initiation times (when the mouse was first moved) were early, and they did not differ between congruent (*M* = 196 ms) and incongruent (*M* = 200 ms) trials (*p* = .22). This ensures that hand movements were on-line with the categorization process and initiated in similar fashion across conditions. Response times (when the response was clicked) also did not differ between congruent (*M* = 1169 ms) and incongruent (*M* = 1171 ms) trials (*p* = .61).

**Figure 3:**
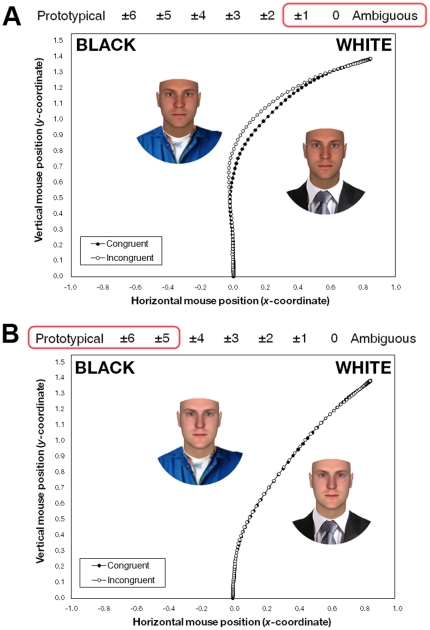
Mean computer mouse trajectories. In the figures, trajectories for all targets were remapped rightward, with the other, unselected race category on the left and the selected race category on the right. A sample face stimulus, surrounded by a status cue associated with the selected race category, is shown on the right, next to the mean trajectory for congruent trials (when faces with high-status cues were categorized as White or faces with low-status cues were categorized as Black). On the left is shown that same face stimulus, but with a status cue associated with the other, unselected race category, next to the mean trajectory for incongruent trials (when faces with high-status cues were categorized as Black or faces with low-status cues were categorized as White). During an actual trial, a single face was centered at the bottom of the screen. As shown in Fig. 2B, trajectories for incongruent trials showed an attraction toward the other race category response, relative to trajectories for congruent trials, and this attraction grew as racial increased in ambiguity. This is exemplified by the difference between the two panels. Panel **A** shows trajectories averaged across trials for the most ambiguous faces (morphs 0 and ±1), along with a sample ambiguous face stimulus. Panel **B** shows trajectories averaged across trials for the least ambiguous faces (morphs ±5 and ±6), along with a sample unambiguously White face stimulus.

To index the degree to which the hand was simultaneously attracted to the other race category on the opposite side of the screen, we computed Area Under the Curve (AUC): the area between the observed trajectory and an idealized straight-line trajectory between the start and endpoints. We regressed trajectories' AUC values onto racial ambiguity, congruency (−0.5 = congruent, 0.5 = incongruent), and the interaction (using normal regression). As expected given prior work [18], there was a significant effect of racial ambiguity. Increases in ambiguity overall led to increases in the attraction toward the opposite side of the screen [*B* = 0.80, *p*<.0001, *z* = 9.03], suggesting that perceivers were tentatively considering the other race category. More importantly, there was a significant effect of congruency. When categorization responses were incongruent (i.e., not influenced by the status cue), hand trajectories nevertheless showed an attraction toward the other race category stereotypically associated with the status cue, relative to hand trajectories for congruent responses, *B* = 0.07, *p*<.01, *z* = 2.73 (Fig. 3). Moreover, a significant interaction indicated that the hand's attraction toward the other race category, due to the presence of a status cue tied to that category, became increasingly strong as racial ambiguity increased, *B* = 0.16, *p*<.05, *z* = 2.07 (Fig. 2B). Thus, en route to settling into the White response for a face with low-status attire, the hand showed an attraction to select the Black response; and en route to settling into the Black response for a face with high-status attire, the hand showed an attraction to select the White response. Further, this attraction effect grew stronger as racial ambiguity increased. We also included initiation time and response time as covariates in the model and reran analyses. This had a negligible effect on the results, confirming that the attraction effect was not a spurious product of differences in the onset or duration of movement. Rather, it reflected a genuine “pull” toward the other race category due to the processing of a status cue associated with that category.

### Simulation

**Figure 4:**
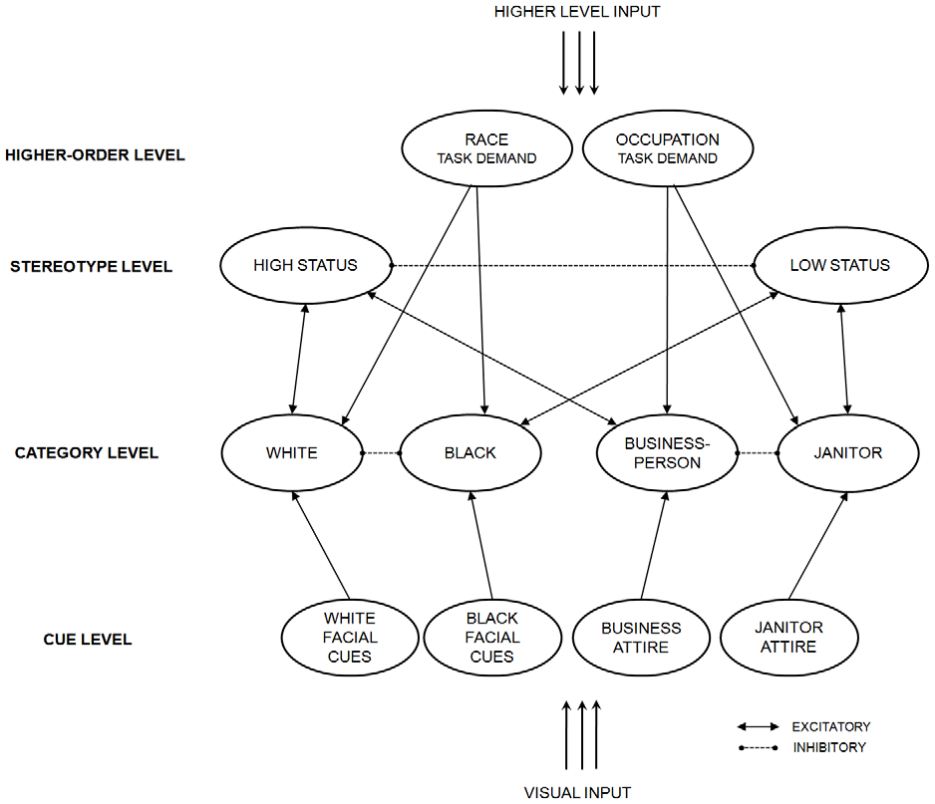
A new instantiation of the dynamic interactive model of person categorization (see [**14**]), a recurrent connectionist network that was used to account for the empirical data.

To more rigorously examine the underlying processing that hypothetically gave rise to the pattern of categorization responses and hand-movement data above, we implemented a simulation of the results using a new instantiation of the computational model described in [14]. The model is a recurrent connectionist network with stochastic interactive activation [26], [27], depicted in Fig. 4. It provides an approximation of the kind of processing that might take place in a human brain [21], [26], [28], [29], specifically in the context of perceiving other people.

Initially, the network is stimulated simultaneously by both visual input and higher-level input. Visual input originates from the visual system, which receives an incoming face stimulus, and higher-level input originates from a top-down attentional system, which directs attention toward race categories (White and Black) or occupation categories (Businessperson and Janitor) based on memory of task instructions (in this case, to categorize race). The network contains a variety of nodes, which have a transient level of activation at each moment in time. This activation corresponds with the strength of a tentative hypothesis that the node is represented in the input. Once the network is initially stimulated, activation flows among all nodes simultaneously as a function of their connection weights. Because many connections between nodes are bidirectional, this flow results in a continual back-and-forth of activation between many nodes in the system. As such, nodes in the system continually readjust each other's activation and mutually constrain one another to find an overall pattern of activation that best fits the inputs. Gradually, the flows of activation lead the network to converge on a stable, steady state, where the activation of each node reaches an asymptote. This final steady state corresponds to an ultimate categorization of another person.

**Figure 5:**
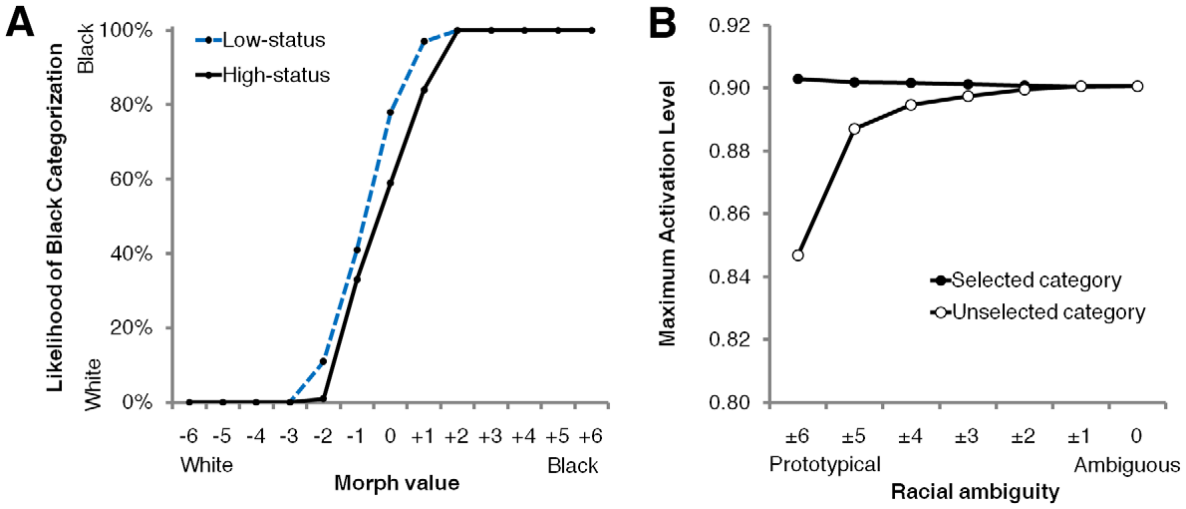
Results of the computational simulations. (**A**) The proportion of simulation runs in which the network settled into the Black category (the Black category node won the competition) after presented with faces that varied along a continuum of race and with either high-status or low-status attire. Note the close correspondence with the data from human perceivers (Fig. 1B). (**B**) Maximum activation level of the winning White or Black category node (the selected category) and the losing White or Black category node (unselected category) is plotted for incongruent trials (when the Black category won the competition for a face with high-status attire, or the White category won the competition for a face with low-status attire), at varying levels of racial ambiguity. Note that the unselected category is partially activated as well, and that as racial ambiguity increases the maximum activation level of the other, unselected category increases correspondingly. This accounts for why participants' mouse movements exhibit an attraction toward that unselected race category, and increasingly so as race becomes more ambiguous (Fig. 3).

When the network was presented with the task demand of race categorization and the face stimuli of Experiments 1 and 2, its categorization responses closely mirrored that of human perceivers (*R* ^2^ = 0.99, root mean-square-error = 0.03; based on human data presented in Figs. 1B and 1C). As shown in Fig. 5A, low-status cues made a Black categorization more probable, whereas high-status cues made a White categorization more probable. Further, these influences of status cue grew stronger as racial ambiguity increased. For those categorization responses that were not affected by the status cue (incongruent responses), the processing of the status cue nevertheless triggered the partial parallel activation of the other race category with which it was associated. This is reflected in Fig. 5B, showing the maximum activation level of the selected and unselected race-category nodes. When a status cue stereotypically tied to the other race category was present (i.e., incongruent trials), that other, unselected category was partially active in parallel. Further, this partial activation of the unselected race category became increasingly strong as racial ambiguity increased. Such partial activation accounts for why participants' hand movements were simultaneously attracted toward the other race-category response (Fig. 3), and why that attraction grew increasingly strong as racial ambiguity increased (Fig. 2B).

Consider, for example, the presentation of a relatively unambiguous White face with janitor attire. A process is set into motion where visual input of the face activates cue nodes and higher-level input of the task demand activates higher-order nodes (see Fig. 4). Activation of the Race Task Demand node starts exciting the White and Black categories and inhibiting the Businessperson and Janitor categories, leading the race categories to become partially active for the task. Strong activation of the White Facial Cues node places strong excitatory pressure on the White category. With both race categories simultaneously active, they begin competing with one another through mutual inhibition to stabilize onto just one. As the competition unfolds, the White category excites the High Status stereotype and the Black category excites the Low Status stereotype. Now with the conflicting Low Status and High Status stereotypes simultaneously active as well, they too begin competing with one another through mutual inhibition to stabilize onto just one. Meanwhile, activation of the Janitor Attire node excites the Janitor category and inhibits the Businessperson category (but the Janitor category only gains a meager amount of activation because it is inhibited by the Race Task Demand node). Ongoing activation of the Janitor category then excites the Low Status stereotype. At this point, the stereotype nodes are being continually fed activation by both race and occupation categories. Because activation in the network is mutually interactive, however, while the competition is still resolving itself the stereotype nodes also feed activation back to the category nodes. This leads the Janitor category's excitation of the Low Status stereotype, in turn, to place excitatory pressure on the Black category and help it win against the White category.

In some cases, such pressures would be strong enough to make the Black category more likely to win the competition, driving ultimate categorization responses. In other cases, such pressures would not be strong enough to drive responses and would only lead to a stronger partial parallel activation of the Black category (until it gradually decays, yielding to the White category). Moreover, these top-down pressures from stereotypes would be given increasingly more room to shape race-category activation as a face's race increases in ambiguity. The lack of bottom-up bias toward either the White or Black category would open the door wider and wider to top-down influences, as the race-category competition is increasingly swayed by feedback from stereotype nodes. By activating stereotype nodes, contextual attire cues readily influence race categorization. As such, the network used status cues to categorize a face's race, particularly when race was ambiguous.

## Discussion

Low-status cues presented with a face increased the likelihood of Black categorization, whereas high-status cues presented with a face increased the likelihood of White categorization. Further, such influences grew stronger as a face's race became more ambiguous, as the bottom-up ambiguity opened the door to top-down pressures from stereotypes triggered by contextual status cues. Often these influences affected categorization wholesale and drove ultimate responses (Experiment 1). In cases where they did not, however, they nevertheless influenced categorization. Even when faces with low-status attire were categorized as White or faces with high-status attire were categorized as Black, the processing of a status cue still triggered the partial parallel activation of the other race category with which it was stereotypically associated. This was evidenced by participants' hands temporarily gravitating toward the other race-category response before arriving at their ultimate categorization (Experiment 2). When status cues do not shape an ultimate categorization, therefore, they nevertheless exert a subtle influence by activating the other, associated race category. Finally, computational simulations demonstrated that these effects were accounted for by a neurally plausible person categorization system, where contextual cues come to trigger stereotypes that in turn exert a top-down influence on race perception.

Social scientists for decades have emphasized the socially constructed nature of race, yet the process by which people are race-categorized in everyday interactions has remained elusive. Recent research in sociology has documented the role that biographical markers of status—like knowledge of another's income level—play in how race is assigned to an individual, noting how the concept of race depends in part on self-fulfilling stereotypes (e.g., [6], [7]). Recent research in psychology, on the other hand, has examined how physical cues drive the race categorization process and bear interpersonal consequences (e.g., [2], [9], [18], [30], [31]). The present studies help unify these two literatures by demonstrating how social stereotypes interact with physical cues to shape the perception of another's race.

In doing so, this work bolsters evidence that higher-order social cognition can constrain lower-level visual processes, and furthers the emerging perspective that perception is driven by an intimate interplay between both sensory and social phenomena [32], [33]. Specifically, the findings support the view that the perception of other people is driven by a dynamic interactive process. In this process, an ongoing and mutually-constraining interaction between various information sources (e.g., bottom-up facial and contextual cues, top-down stereotypes) triggers partially-active categories that, over time, settle into a stable and integrated perception [14]. As such, person perception readily makes compromises between how other people “actually” appear and the stereotyped expectations dictating how they “should” appear.

Such malleability in race perception might bear numerous implications for downstream interpersonal phenomena. It has long been known that once race is perceived, it provides a lens for subsequent interaction by molding judgments and impressions and by triggering affective and behavioral reactions [1], [3], [22], [34], [35]. If status cues shape race perception, therefore, they are also likely to shape a variety of downstream phenomena. For example, recent work has suggested that status cues can affect the memory of faces [36]. Although such downstream effects are likely to be particularly important for ambiguous faces (as we find status cues to have the largest influences in such circumstances), our findings suggest that status cues might bear downstream consequences for more racially unambiguous faces as well. This would be due to the partial parallel activation of other race categories. For example, we found that a status cue can trigger the simultaneous activation of a race category with which it is stereotypically associated, although that category is not ultimately perceived. This was evidenced by the hand's partial attraction to the other category (e.g., Black) before settling into an ultimate categorization response (e.g., White). Although such attraction “ended” with a final mouse-click, this is unlikely to be the end of that category's influences, as the lasting consequences of even the subtlest of category activations (e.g., via priming) have been widely documented [1], [3], [22], [34], [35]. Thus, when a target with low-status attire is perceived as White or a target with high-status attire is perceived as Black, the status cue's partial activation of the other race category could potentially trigger a number of downstream social consequences. Future research could directly examine such consequences, as well as how they might be moderated by individual differences in racial prejudice (e.g., see [37]).

The current findings also contribute to a growing body of research examining perceived racial ambiguity and the categorization of multiracial individuals. Previous work has tended to focus on overall patterns in race categorization, such as hypodescent—the tendency to categorize multiracial individuals to a subordinate social group. For instance, studies have investigated how such categorization patterns are affected by internalized group hierarchies [38], by basic cognitive learning processes [39], by motivations and lay beliefs about the nature of race [40], [41], and by more reflexive categorization tasks [23]. The present results demonstrate yet another factor influencing race categorization—stereotypes triggered by contextual cues—and how such influences grow with increasing levels of racial ambiguity. The finding that ambiguity renders categorizations more susceptible to stereotypes is particularly noteworthy given that the number of multiracial Americans is likely to continue to increase over the next several decades [42].

The results also serve to advance current theoretical models of person categorization more generally. In Experiment 2, for example, we used mouse-tracking to flesh out the temporal dynamics through which bottom-up face processing interacts with top-down social factors (stereotypes in the present case, but perhaps extendable to other factors as well, e.g., prior knowledge of group hierarchies, motivations, lay beliefs). Previous mouse-tracking work showed that race categorization involves a dynamic process of partially-active categories competing over time—a competition that is exacerbated with increasing levels of racial ambiguity [18], [19]. Here, we showed that this competition process is continuously weighed in on not only by bottom-up facial cues, but also by other information sources, such as top-down stereotypes. For example, we found that when categorizing faces with low-status attire as White or categorizing faces with high-status attire as Black, mouse movements were neither in a discrete pursuit straight toward the White response, nor in a discrete pursuit straight toward the Black response. Rather, as seen with the hand's continuous attraction toward the opposite race-category response in Fig. 3A, at each moment the location of the mouse was in some weighted combination between one pursuit driven by bottom-up face processing (e.g., White) and a simultaneous pursuit driven by top-down stereotypes (e.g., Black), before stabilizing onto an ultimate categorization response (e.g., White). This demonstrates how bottom-up face processing interacts in real-time with top-down social factors, continuously across the construal process. Such evidence furthers current theoretical models, as this continuous top-down interactivity was recently predicted but was until now lacking empirical demonstration [14].

It is also important to note potential limitations of the present work. Although we have suggested that the effects of contextual cues on race categorization were driven by differences in the social status of occupations, it is possible that they were driven by other differences related to occupations, such as stereotypes of intelligence, pay, or who does “dirty work” (e.g., [43], [44]). Thus, the present effects might not be direct effects of social status, but could potentially be accounted for by other characteristics that are related to it. In either case, the results show how status-related stereotypes activated by contextual cues readily exert a top-down influence on race perception. In addition, there are some limitations to defining a face's racial cues using the statistical face model we adopted (see Materials & Methods) [45]. The model has been used by many researchers to generate faces varying along a number of dimensions, but the extent to which the model's dimensions can be considered representative of the U.S. population has yet to be examined. Finally, while the current work examined how a particular instance of the race categorization process settles into an ultimate stable category over hundreds of milliseconds, we do not wish to imply that the category would necessarily be fixed over longer periods of time, such as months or years (e.g., [6]). Quite the opposite, our results suggest that race categorization is a highly malleable process and readily influenced by the context.

In summary, although it is commonly believed that race is perceived through another's facial features, we have shown that there lies much beyond the face that shapes perception. Social status cues that often surround a face in the real world, such as attire, systematically changed the perception of a face's race. Across two experiments and a series of computational simulations, we demonstrated how stereotypes flexibly interact with physical cues to shape race perception. Sometimes, a status cue activated stereotypes that influenced categorization wholesale, changing how a face was ultimately perceived. Other times, it activated stereotypes that influenced categorization only subtly, by simultaneously activating another race category (associated with the status cue) and temporarily shifting race perception. Thus, although racial prejudice is often thought to be a consequence of initially categorizing others [46], here we show that our prejudices affect even initial categorization, highlighting how social and contextual factors shape basic person construal.

## Materials and Methods

All research involving human participants was approved by the Institutional Review Board at Tufts University, and informed consent was obtained from all participants.

### Experiment 1

Thirty-four undergraduates participated for partial course credit or monetary compensation. The mean age of participants was 21.3, and 13 were male. 26 self-identified their race as White, 1 as Black, 3 as East Asian, 3 as South Asian, and 1 as Biracial. Participants were presented with faces in a randomized order and asked to categorize them as White or Black using the keyboard as quickly and accurately as possible. Face stimuli were comprised of 16 computer-generated face identities (8 male) that were morphed along a 13-point race continuum, from White (morph −6) to Black (morph +6), using FaceGen Modeler. This software generates faces using a statistical 3D model based on anthropometric parameters derived from laser scans of human faces. The model does not make assumptions about what differs between White and Black faces; rather, by averaging across many faces, parameters that emerge as reliably different between races are incorporated into the morphing algorithm [45]. Parameters that do not differ systematically are held constant. Using various images of clothing obtained from public domain websites, each face was affixed to a high-status (business) and low-status (janitor) attire (Fig. 1A). Half of the 16 face identities (each containing 13 levels of race) were affixed to a high-status cue, whereas the other half were affixed to a low-status cue (which identities were affixed to which cue was counterbalanced across participants).

### Experiment 2

Twenty-two undergraduates participated for partial course credit or monetary compensation. The mean age of participants was 20.4, and 11 were male. 16 self-identified their race as White, 2 as Black, 3 as East Asian, and 1 as South Asian. One participant did not follow instructions correctly, leaving 21 participants for analysis. Participants categorized the same face stimuli used in Experiment 1, except here in a mouse-tracking paradigm. On every trial, participants clicked a “Start” button located at the bottom-center of the screen, which was then replaced by a face. Faces were presented in a randomized order and categorized by clicking either a “White” or “Black” response located in the top-left and top-right corners of the screen (which category appeared on the left vs. right was counterbalanced across participants). Meanwhile, we recorded the streaming *x*, *y* coordinates of the computer mouse (sampling rate≈70 Hz). To ensure trajectories were on-line with the categorization process, we encouraged participants to begin initiating movement early. As in previous research (e.g., [18]), if participants initiated movement later than 400 ms following face presentation, a message appeared after the trial encouraging them to start moving earlier on future trials. To record and analyze mouse trajectory data, we used the freely available MouseTracker software package: http://mousetracker.jbfreeman.net [47].

Once collected, all mouse trajectories were rescaled into a standard *x*, *y* coordinate space: top-left [1, 1.5] and bottom-right [1, 0], leaving the start position of the mouse at [0, 0]. Trajectories were normalized (linearly interpolated) into 101 time steps (100 time bins) to permit averaging of their full length across multiple trials. For comparison, all trajectories were remapped rightward, such that the selected response was at the top-right and the unselected response at the top-left. To obtain a by-trial index of the degree to which the mouse was attracted toward the other racecategory (indexing the simultaneous activation of that category), we computed Area under the Curve (AUC): the area between the observed trajectory and an idealized response trajectory (a straight line between the trajectory's start and endpoints). See [47] for further details on mouse trajectory preprocessing and analytic techniques.

### Simulation

In the model, how the activation of a node changes over time is determined by three factors: the node's prior activation, how quickly the activation decays, and the net input of activation into the node from other nodes. On each iteration, excitation and inhibition summate algebraically to determine the net input to a node. The net input is also altered by normally distributed noise as well as any external input into the node. Before the presentation of each face stimulus, activations of all nodes in the network are set equal to a resting activation value, and external inputs are presented to certain nodes for processing. Processing occurs over a number of iterations. On each iteration, each node computes its net input from the nodes connected to it based on their latest activation. Specifically, the net input to node *i* is:
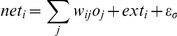
where *w_ij_* is the connection weight to node *i* from node *j*, *o_j_* is the greater of 0 and the activation of node *j*, *ext_i_* is any external input to node *i*, and ε_σ_ is a small amount of normally distributed random noise with mean 0 and standard deviation σ. Once the net input into all nodes has been computed, the activation of node *i* is updated as:
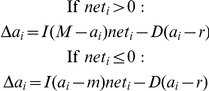
such that *M* is the maximum activation, *m* is the minimum activation, *r* is the resting activation level, *I* is a constant that scales the influence of external inputs on a node, and *D* is a constant that scales a node's tendency to decay back to rest. The parameters are as follows: *M* = 1, *m* = −0.2, *r* = 0, *I* = 0.4, *D* = 0.1, and σ = 0.007. See [14] for complete details on the general model. In this instantiation, all excitatory connections have a weight of 0.2 and inhibitory connections a weight of −0.1. Because non-normative categories (e.g., Black) and the stereotypes tied to those categories (e.g., low-status) tend to be more readily activated [48], and because race categorization tends to be more strongly swayed by the Black category rather than the White category (e.g., [23]), the bidirectional excitatory Black–Low-Status connection was given a slightly stronger weight of 0.203, thereby capturing this asymmetry. Network parameters, connection weights, and input values were set based on our prior studies, intuitions regarding stimulus and task features, and previous simulations with this model [14].

We conducted 26 simulations: 13 morph values×2 status cues. In each simulation, input into the Race Task Demand node was set at .9 and into the Occupation Task Demand node at .1, simulating the task demand that requires attention on race rather than occupation. For the high-status condition (where targets had business attire), we set input into the Business Attire node at 1 and into the Janitor Attire node at 0, and vice-versa for the low-status condition (where targets had janitor attire). Based on a face's morph value, we set input into the White Facial Cues node at [1−(morph+6)/12] and input into the Black Facial Cues node at [(morph+6)/12]. For example, for the most prototypically White face (morph −6), the White Facial Cues node was initialized with 1 and Black Facial Cues node with 0, and vice-versa for the most prototypically Black face (morph +6). For a slightly less White face (morph −5), the White Facial Cues node was initialized with 0.92 and the Black Facial Cues node with 0.08. For the most racially ambiguous face (morph 0), both nodes were initialized with 0.5. We ran each of the simulations 100 times. After 200 iterations, we selected the race-category node with the highest activation as the network's categorization response.

Note that the present instantiation of the model is a simpler variant than previous instantiations of the model [14], in that there are less between-node connections. A more complex instantiation (Fig. S1) was also used, but because the simpler instantiation also was able to account for the empirical data well, it was adopted for parsimony.

## Supporting Information

Figure S1 **Another version of the computational model used, which contains a more complex arrangement of between-node connections.** The simpler model shown in Fig. 4 was adopted for parsimony.
(TIF)
