# Supplementary figures and images for: Looking the Part: Social Status Cues Shape Race Perception
Source: PLoS One. 2011 Sep 26;6(9):e25107. doi: 10.1371/journal.pone.0025107 (PMC3180382; doi:10.1371/journal.pone.0025107)

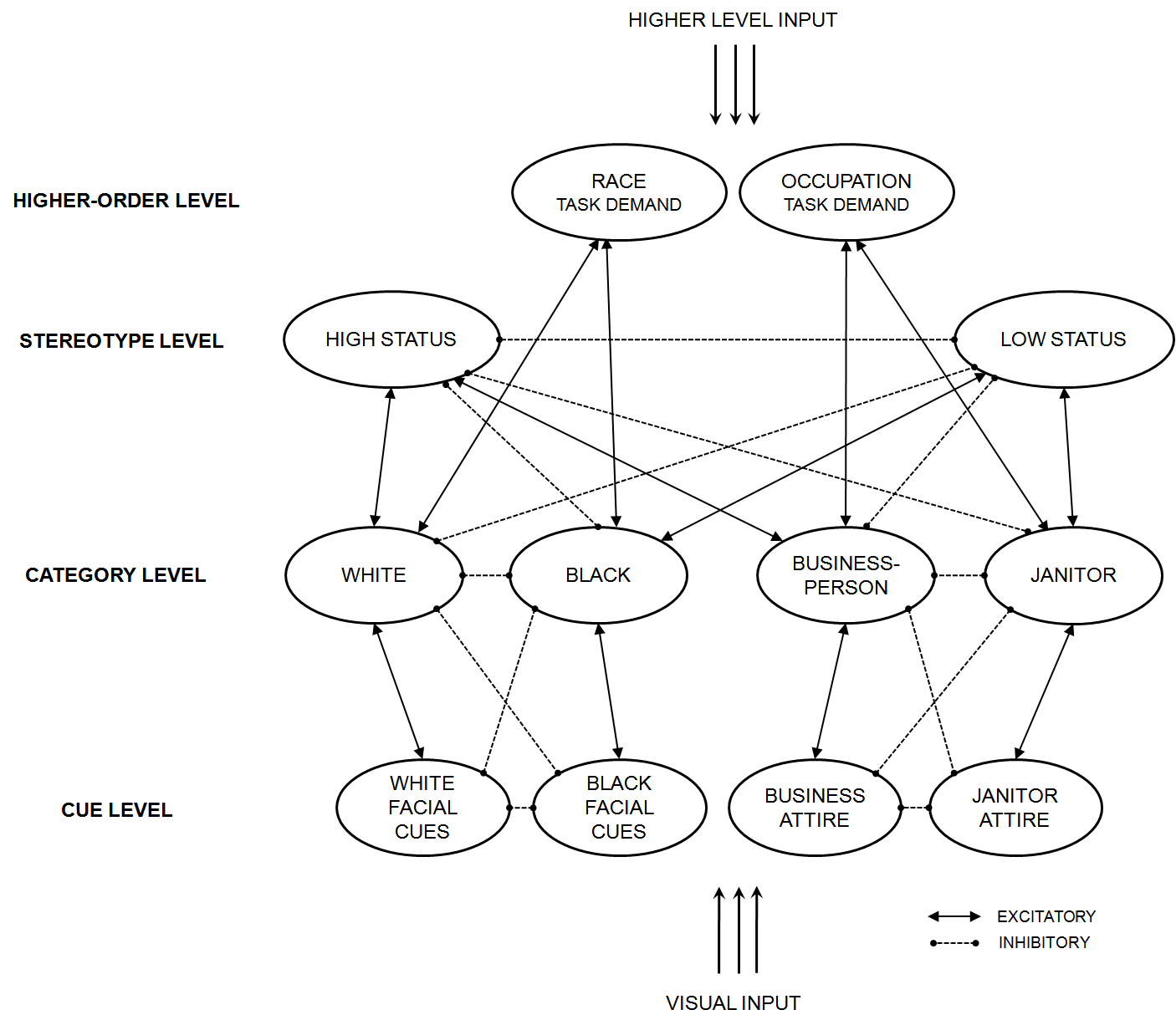

Supplement: Figure S1 — Another version of the computational model used, which contains a more complex arrangement of between-node connections. The simpler model shown in Fig. 4 was adopted for parsimony. (TIF) [file pone.0025107.s001.tif]
